# Comparison of a Standardized High-Fat Meal versus a High-Fat Meal Scaled to Body Mass for Measuring Postprandial Triglycerides: A Randomized Crossover Study

**DOI:** 10.3390/metabo12010081

**Published:** 2022-01-15

**Authors:** Bryant H. Keirns, Christina M. Sciarrillo, Samantha M. Hart, Sam R. Emerson

**Affiliations:** Department of Nutritional Sciences, 208 Nancy Randolph Davis, Oklahoma State University, Stillwater, OK 74078, USA; bryant.keirns@okstate.edu (B.H.K.); csciarr@ostatemail.okstate.edu (C.M.S.); snielso@ostatemail.okstate.edu (S.M.H.)

**Keywords:** postprandial triglycerides, high-fat meals, cardiovascular disease, risk screening, dyslipidemia, blood lipids, metabolic testing

## Abstract

Post-meal triglycerides are an independent cardiovascular disease (CVD) risk factor, but the ideal high-fat meal formulation has yet to be standardized and is one challenge prohibiting widespread clinical adoption of postprandial triglyceride assessment. Two general approaches often used are giving individuals a high-fat meal scaled to body weight or a standardized high-fat meal containing a set fat bolus. A recent expert panel statement has endorsed the latter, specifying 75 g of fat as an appropriate fat dosage. Despite this recommendation, no study to date has tested whether there is a difference in postprandial triglycerides or if risk classification is affected based on these different approaches. We recruited 16 generally healthy individuals with roughly equal distribution among body mass index (BMI)class (*n* = 5–6/per BMI category) and sex (*n* = 2–3 M/F) within each BMI class. Each participant underwent two abbreviated fat tolerance tests separated by ~1 week: one with a scaled to body weight high-fat meal (9 kcal/kg; 70% fat) and a standardized meal containing 75 g of fat (70% fat). Fasting, 4 h, and absolute change in triglycerides across the entire sample and within each BMI category were similar regardless of high-fat meal. Only one participant with obesity had discordant postprandial responses between the fat tolerance tests (i.e., different CVD risk classification). These findings suggest that, within a certain range of fat intake, generally healthy individuals will have a similar postprandial triglyceride response. Considering the greater convenience of utilizing standardized high-fat meals, our data suggest that a standardized high-fat meal may be acceptable for large-scale studies and clinical implementation.

## 1. Introduction

Interest in post-meal triglycerides as a cardiovascular disease (CVD) risk factor has grown in recent years, largely due to the finding that nonfasting triglycerides (i.e., triglycerides measured within 8 h of eating any meal) are an independent CVD risk factor [1,2]. A similar measurement—postprandial triglycerides (i.e., serial triglyceride measurements after an administered high-fat meal)—is more controlled than nonfasting triglycerides and thus may be preferred [3].However, postprandial protocols are accompanied by other challenges including long assessment periods in research laboratories and lack of consensus as to whether high-fat meals should be scaled to body weight or administered as a set fat bolus.

While work has been done to validate an abbreviated fat tolerance test [4,5], there is still not agreement regarding the most appropriate high-fat meal formulation, as scaling high-fat meals and providing a set fat bolus each has its own advantages and disadvantages. For example, a standardized fat bolus would be easier to utilize in large epidemiological studies and potentially allow for an official reference range to be set for postprandial triglycerides (as has been done with postprandial glucose in oral glucose tolerance tests). These benefits have led to an expert panel on nonfasting and postprandial triglycerides to recommend a standardized meal containing 75 g of fat for assessing postprandial triglycerides [6]. However, a large fat bolus may have a saturating effect in individuals with lower body weight (i.e., dietary triglyceride accumulating faster than it can be hydrolyzed), such that the inability to clear dietary triglyceride would falsely suggest increased CVD risk. Conversely, with a high-fat meal scaled to body weight, there is little risk of saturating triglyceride clearance mechanisms of those with lower body weight, but other concerns exist. For instance, it is possible that individuals with obesity will have artificially elevated triglycerides compared to normal-weight individuals that is driven by higher fat intake with a scaled to body weight meal, not underlying metabolic disturbances that contribute to postprandial hyperlipidemia.

Despite this disagreement over high-fat meal composition, no study to date has directly tested whether there is a measurable difference in postprandial triglycerides when consuming a high-fat meal administered as a standardized bolus versus scaled to body weight or if a given individual would change risk categories per current guidelines (i.e., above or below 220 mg/dL) depending on differences in high-fat meal formulation [6]. Therefore, the aim of the present study was to determine whether the postprandial triglyceride response differed when the same individuals consumed a meal scaled to body weigh high-fat versus a fat bolus of 75 g (as has been recommended by an expert panel on postprandial triglycerides) in the context of an abbreviated fat tolerance test across a range of body mass indexes (BMI).

## 2. Results

**Figure 1:**
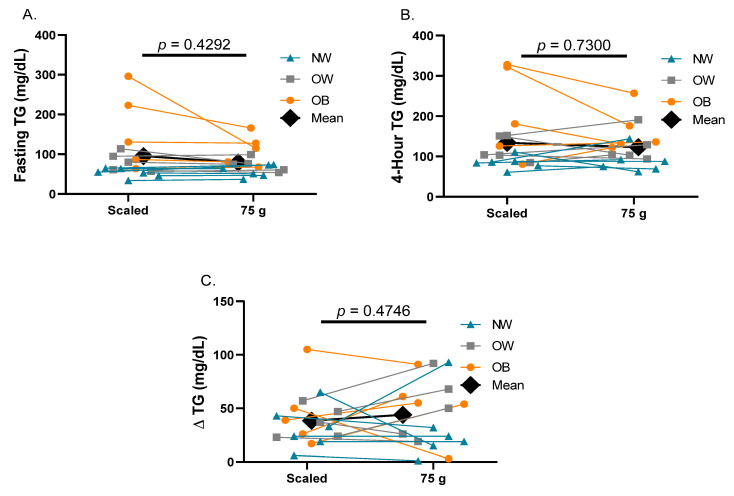
**Postprandial Triglyceride Responses to a Scaled to Body Weight versus 75 g High-fat Meal**. (**A**) Fasting triglycerides, (**B**) 4 h triglycerides, and (**C**) Change in triglycerides in the scaled-to-body-weight and 75 g fat tolerance tests. Each pair of connected data points represents the same individual at each study visit. Data are presented as mean ± SD. Abbreviations: NW normal weight; OW overweight; OB obese; TG triglycerides.

Participant characteristics are presented in Table 1. Participants had similar fasting triglycerides for both fat tolerance tests (*p* = 0.43; Figure 1A). Additionally, 4 h triglycerides (Figure 1B) and the absolute change in triglycerides (Figure 1C) were similar after participants consumed the scaled to body weight and the 75 g high-fat meals (*p* values ≥ 0.47). When comparing fasting, 4 h, and change in triglycerides between the two high-fat meals within each BMI class, no differences were observed for any triglyceride parameter (*p* values ≥ 0.19; Table 2). Only one participant in the obese BMI group had discordant postprandial responses (scaled 4 h triglycerides = 322 mg/dL, 75 g 4 h triglycerides = 176 mg/dL).

Subjective hunger was assessed to evaluate participants’ tolerance of the 75 g high-fat shake, which was higher in fat than the shake scaled to body weight for 14 of 16 participants. Satiety was similar after the scaled shake and 75 g shake (*p* = 0.21; scaled = 44.1 ± 34.2 a.u.; 75 g = 47.4 ± 44.9 a.u.; data not shown), and on average participants fell closest to the “moderately full” category.

## 3. Discussion

This study was motivated by ongoing disagreement about the most appropriate high-fat meal formulation to use when testing postprandial triglycerides. Indeed, a wide variety of high-fat meal preparations are utilized in research studies, including commercial products and prepared mixed meals both as a set quantity and scaled to body weight (e.g., [7,8,9])These differences in high-fat meal formulation make comparing results across studies difficult and have led an expert panel to recommend a standardized fat tolerance test containing 75 g of fat for postprandial triglyceride testing [6].Despite this recommendation, to our knowledge, no study has examined if there is a measurable difference in postprandial triglycerides when consuming a shake scaled to body-weight versus a 75 g fat bolus or if the amount of fat consumed would lead to the same individual changing risk categories.

In the present study, we observed that 4 h triglycerides and the absolute change in triglycerides were similar after the scaled to body weight high-fat meal (9 kcal/kg) and the standardized shake containing 75 g of fat across our entire sample. Similarly, there were no differences when analyzing each BMI class individually. Currently, >220 mg/dL has been put forth as the only guideline for an adverse postprandial triglyceride response by an expert panel (other organizations only have nonfasting cutoffs) [6]. Only one male participant in the group with obesity changed risk categories depending on the high-fat meal consumed. However, given that this participant had lower 4 h triglycerides after the 75 g shake (consumed 61 g of fat with scaled shake), this observation does not appear to be related to acute fat intake and is likely due to other factors (e.g., increased triglyceride variability associated with males and higher baseline triglycerides, variation in lifestyle factors between sessions) [10]. Moreover, this participant presented with other CVD risk factors throughout the study such as fasting glucose of 105 mg/dL and fasting triglycerides of 296 mg/dL on separate occasions. Therefore, postprandial triglyceride testing may not provide much additional insight in a real-world setting for this participant. Within individuals with a normal BMI, all 4 h triglyceride measurements were well below 220 mg/dL regardless of the high-fat meal consumed, indicating that the 75 g shake did not overwhelm triglyceride clearance by lipoprotein lipase in normal-weight individuals and falsely imply CVD risk. Overall, these findings suggest that similar results can be expected when using the shake scaled to weight used herein and a bolus containing 75 g of fat.

Our data have several implications for postprandial triglyceride testing. For one, in a population without overt cardiometabolic disease, a 75 g fat bolus may provide roughly the same information about CVD risk as a scaled-to-weight high-fat meal, and it was generally well-tolerated. This data should allow for easier interpretation and synthesis of current research that have used a variety of high-fat meal formulations. Additionally, individuals with lower body mass do not appear to change risk categories when consuming an oral fat challenge between ~40 to 75 g of fat, meaning that a 75 g shake could be used at the population level without concern of inappropriate risk classification. When taking these observations together, and when considering that it would be much simpler to mass produce standardized high-fat meals, it may be advantageous to use a standardized high-fat meal similar to what was utilized here in future epidemiological studies to move towards establishing official reference ranges for postprandial triglycerides.

The present study possesses strengths as well as limitations. Strengths of the study include that we recruited individuals in the normal-weight, overweight, and obese BMI categories, making our results more generalizable. In addition, the high-fat meal utilized here is hypoallergenic, inclusive (i.e., can be consumed by vegetarians/vegans), and simple to make and thus could be used at scale. Limitations include that, while the abbreviated fat tolerance test utilized has been validated against serial postprandial triglyceride measurement for six hours, it is still possible that we may have missed peak postprandial triglycerides in some individuals by only measuring fasting and 4 h triglycerides. Additionally, this abbreviated test was validated with the shake scaled to body weight used in this study, not the standardized 75 g shake. Lastly, we acknowledge that our data were collected in a group of disease-free, primarily Caucasian individuals, so this work should be repeated in other ethnic groups and validated in disease states, as needed. Overall, we conclude that in individuals free of overt cardiometabolic disease, a scaled-to-body-weight high-fat meal and a standardized meal containing 75 g of fat yield a similar postprandial triglyceride response.

## 4. Materials and Methods

### 4.1. Participants

Participants were recruited through mass email, word of mouth, and the snowball method at the Oklahoma State University Stillwater Campus. Sixteen total participants between the ages of 18 and 45 were recruited, with roughly even distribution among the normal-weight (18.5–24.9 kg/m^2^), overweight (25.0–20.9 kg/m^2^), and obese BMI categories (>30.0 kg/m^2^; *n* = 5–6/per BMI category) and sex (*n* = 2–3 M/F) within each BMI class. No participants had muscle mass greater than 35%. Initial exclusion criteria were having an existing cardiometabolic condition, being pregnant, and using lipid-lowering drugs, anti-hypertensives, tobacco products, and/or illicit drugs. Participants meeting these criteria were invited to an in-laboratory initial assessment. The study was carried out according to the Declaration of Helsinki and approved by the Oklahoma State University Institutional Review Board (IRB-21-279-STW, approved 21 July 2021).

### 4.2. Initial Assessment

The initial visit consisted of informed consent and blood pressure (Omron 5 Series BP742N; Kyoto, Japan), general anthropometric, and body composition (Seca mBCA 514; Hamburg, Germany) measurements. Participants were then randomized to undergo two fat tolerance tests in a crossover design, where they consumed both a scaled-to-body-weight high-fat shake and a standardized high-fat shake (more details below). The two fat tolerance tests were separated by approximately one week and started between 0630 and 0930 h.

### 4.3. Fat Tolerance Tests

Prior to each fat tolerance test, participants were instructed to abstain from alcohol and exercise for 24 h. Additionally, participants reported having fasted for 10 h and consumed the same snack before initiating their fast. Participants completed a 3-day food record before each visit and were asked to keep their overall diet consistent between the two fat tolerance tests. An abbreviated fat tolerance test was utilized for both visits where fasting triglycerides were measured (more details below), a high-fat meal was consumed (either scaled to weight or a standardized bolus), and then triglycerides were measured again 4 h post high-fat meal. Between blood draws, participants were free to leave the laboratory, but did not eat or drink anything other than water and avoided exercise. This abbreviated fat tolerance test is valid and reliable compared to postprandial triglyceride protocols where triglycerides are measured hourly for six hours [4,5]. The meal scaled to body weight was administered at 9 kcal/kg body mass (70% fat, 20% carbohydrate, 10% protein), and the standardized meal contained 75 g fat, 51 g carbohydrate, 24 g protein. To prepare both high-fat meals, commercially available coconut cream (3.1 mL/kg), chocolate syrup (0.4 g/kg), and pea protein powder (0.4 g/kg) were combined in blender and mixed for ~60 s. Both high-fat meals were administered at this relative macronutrient distribution; the only difference was in the ingredient quantities. The above ingredient quantities were added until a given participant’s body weight was reached for the scaled high-fat meal. The 75 g high-fat meal corresponded to a 107 kg individual when using the same ingredient quantities per kg. The 75 g high-fat meal is similar to what has been recommended by an expert panel for postprandial triglyceride testing [6].

Serum total-cholesterol (total-C), high-density lipoprotein cholesterol (HDL-C), triglycerides, glucose, alanine transaminase (ALT), and aspartate aminotransferase (AST) were measured directly with the Piccolo Xpress on Lipid Panel Plus reagent discs (Abbot; Chicago, IL, USA). Each reagent disc conducts an internal quality control check. These reagent discs deploy enzymatic methods for measurement of triglycerides (glycerol kinase method), total-C (cholesterol esterase and cholesterol dehydrogenase method), HDL-C (modified cholesterol esterase and cholesterol oxidase method), glucose (hexokinase and glucose oxidase method), ALT (ALT and lactate dehydrogenase method), and AST (AST and malate dehydrogenase method). Low-density lipoprotein cholesterol (LDL-C) was calculated using the Friedewald equation. Very low-density lipoprotein cholesterol (VLDL-C) was calculated by dividing triglycerides by 5. Non-HDL-C was calculated by subtracting HDL-C from total-C. CVs for all lipids and glucose are 1–3%. CVs for ALT and AST were 6% and 3%, respectively. The reference intervals for measured enzymes were as follows: ALT (10–47 U/L), AST (11–38 U/L).

Subjective satiety after each fat tolerance test was assessed using the Satiety Labeled Intensity Magnitude scale (SLIM) survey [11]. The SLIM allows individuals to select a satiety score from −100 (greatest imaginable hunger) to 100 (greatest imaginable fullness).

### 4.4. Statistical Analyses

Data were first tested for normality using the Shapiro–Wilk test. Normally distributed data were analyzed using paired *t*-tests. Data not fitting the normal distribution were analyzed with Wilcoxon’s signed-rank test. All data were analyzed with GraphPad Prism 8.0 (La Jolla, CA, USA) and alpha was set at 0.05.

## Figures and Tables

**Table 1:** Participant and Meal Characteristics.

	All (*n* = 16)	NW (*n* = 6)	OW (*n* = 5)	OB (*n* = 5)
**Participant Characteristics**				
Age (years)	28 ± 7	28 ± 10	29 ± 8	25 ± 6
Mass (kg)	84.5 ± 22.1	68.4 ± 13.8	81.6 ± 9.9	106.7 ± 21.8
BMI (kg/m^2^)	27.3 ± 6.2	21.6 ± 2.6	26.7 ± 0.8	34.7 ± 4.4
WC (inches)	34.5 ± 6.7	28.8 ± 4.1	33.8 ± 3.0	42.2 ± 3.8
Body Fat (%)	29.5 ± 11.4	19.3 ± 3.8	30.3 ± 9.4	40.8 ± 7.7
Muscle mass (%)	34.2 ± 5.5	38.3 ± 3.3	34.1 ± 5.9	29.4 ± 3.8
Systolic BP (mmHg)	120 ± 15	111 ± 10	122 ± 16	126 ± 17
Diastolic BP (mmHg)	80 ± 8	72 ± 3	83 ± 6	84 ± 8
Fasting Glucose (mg/dL)	101.5 ± 7.8	100.0 ± 9.5	105.0 ± 10.8	100.0 ± 3.4
Fasting Total-C (mg/dL)	151.3 ± 26.2	140.0 ± 18.3	153.0 ± 11.5	163.0 ± 40.8
Fasting HDL-C (mg/dL)	51.3 ± 11.5	53.0 ± 9.6	54.4 ± 17.5	46.2 ± 4.9
Fasting LDL-C (mg/dL)	83.8 ± 19.6	76.5 ± 7.4	83.6 ± 15.5	92.6 ± 30.9
Fasting VLDL-C (mg/dL)	16.3 ± 9.1	10.7 ± 2.3	14.8 ± 3.6	24.6 ± 12.5
Fasting Non-HDL-C (mg/dL)	100.1 ± 26.3	87.2 ± 9.1	98.6 ± 16.9	117.2 ± 39.7
Fasting Triglycerides (mg/dL)	81.4 ± 45.1	52.8 ± 11.7	74.8 ± 16.8	122.2 ± 61.9
Fasting ALT (U/L)	27.3 ± 6.4	23.8 ± 4.3	26.3 ± 7.6	31.0 ± 5.7
Fasting AST (U/L)	27.3 ± 6.3	25.5 ± 5.2	30.0 ± 10.0	26.6 ± 3.2
**Scaled High-fat Meal Characteristics**				
Total energy (kcal)	760.4 ± 198.6	615.6 ± 124.4	734.6 ± 89.2	959.9 ± 196.4
Fat (g)	59.2 ± 15.4	47.9 ± 9.7	57.2 ± 6.9	74.7 ± 15.3
Carbohydrate (g)	39.9 ± 10.4	32.3 ± 6.5	38.6 ± 4.7	50.4 ± 10.3
Protein (g)	19.0 ± 5.0	15.4 ± 3.1	18.4 ± 2.2	24.0 ± 4.9

Data are presented as mean ± SD. Abbreviations: **NW** normal weight; **OW** overweight; **OB** obese; **BMI** body mass index; **WC** waist circumference; **BP** blood pressure; **HDL** high-density lipoprotein; **LDL** low-density lipoprotein; **VLDL** very low-density lipoprotein; **ALT** alanine transaminase; **AST** aspartate aminotransferase.

**Table 2:** Postprandial Triglyceride Responses to the Scaled and 75 g High-fat Meals within BMI Category.

	NW (*n* = 6)			OW (*n* = 5)			OB (*n* = 5)		
	Scaled	75 g	*p*	Scaled	75 g	*p*	Scaled	75 g	*p*
Fasting TG (mg/dL)	52.8 ± 11.7	57.7 ± 15.0	0.19	81.4 ± 23.8	73.2 ± 17.3	0.34	160.2 ± 97.3	111.6 ± 39.0	0.24
4 h TG (mg/dL)	84.5 ± 16.3	88.3 ± 29.5	0.80	119.0 ± 30.6	124.2 ± 39.5	0.75	207.6 ± 112.9	164.4 ± 55.7	0.26
Delta TG (mg/dL)	31.7 ± 20.6	30.7 ± 32.2	0.95	37.6 ± 14.7	51.0 ± 30.1	0.21	47.4 ± 34.6	52.8 ± 31.7	0.75

Data are presented as mean ± SD. Abbreviations: **NW** normal-weight; **OW** overweight; **OB** obese; **TG** triglycerides.

## Data Availability

The data presented in this study are available in article.
